# Radial Inertia Effect of Ultra-Soft Materials from Hopkinson Bar and Solution Methodologies

**DOI:** 10.3390/ma17153793

**Published:** 2024-08-01

**Authors:** Yue Liu, Yongshuai Wang, Qiong Deng

**Affiliations:** 1 Joint International Research Laboratory of Impact Dynamics and Its Engineering Applications, School of Aeronautics, Northwestern Polytechnical University, Xi’an 710072, China; 2 Shaanxi Key Laboratory of Impact Dynamics and Its Engineering Application, School of Aeronautics, Northwestern Polytechnical University, Xi’an 710072, China

**Keywords:** split-Hopkinson pressure bar, ultra-soft material, radial inertia effect, spike-like stress feature, finite element analysis

## Abstract

The split-Hopkinson pressure bar technique is widely used to determine the dynamic mechanical behavior of materials. However, spike-like stress features appear in the initial stress behavior of ultra-soft materials tested with a split-Hopkinson bar. These features are not intrinsic characteristics of the materials. Potential causes were investigated through experiments and numerical simulations. It was found that the spike feature represents derived stress resulting from the radial inertia effect during dynamic loading. In this work, we propose and experimentally verify effective methods to reduce this effect. The influences of density, strain acceleration, ratio between inner and outer diameter, and Poisson’s ratio on the radial inertia effect were investigated. The spike stress was found to change linearly with density and strain acceleration but decrease significantly when the inner/outer diameter ratio was below 0.3, after which it remained nearly constant. A parabolic stress distribution was observed along the radial direction due to the Poisson effect, especially when the ratio exceeded 0.3, leading to higher spike stress. Finally, suggestions were proposed as experimental guidance when testing ultra-soft materials.

## 1. Introduction

Ultra-soft materials have low wave impedance and strength, and they usually exhibit high-rate dependency when subjected to dynamic loading, as seen in applications involving biological tissue [1], gels [2], and polymers [3,4], etc. Their mechanical behaviors under high-strain-rate loading differ significantly from those under quasi–static loading, such as material strength [1,2], deformation stage [3,4], and deformation mechanism [5]. Therefore, it is crucial to determine the dynamic mechanical properties of ultra-soft materials for their safe application. The spilt-Hopkinson pressure bar (SHPB) is the most effective device to study materials’ properties at high strain rates between 10^2^ and 10^4^ s^−1^ [6]. The fundamental assumption is that specimen deformation occurs in a dynamic stress equilibrium state. However, for ultra-soft materials with an extremely low wave speed, achieving stress equilibrium loading on the specimen with a common trapezoid stress wave is difficult, leading to experimental errors [7]. Thus, it is necessary to modify an SHPB for ultra-soft material to address experimental difficulties such as weak transmitted signals [8,9], stress equilibrium [10], and constant strain rate [11], to improve measurement accuracy.

Pulse shaping techniques are commonly used to filter out high-frequency components of the incident wave and extend its rising edge time, facilitating a rapid stress equilibrium [12,13,14]. Using thin specimens is an alternative method [15,16] to help achieve a stress equilibrium, which can increase the reverberation order numbers of stress waves across specimens. These techniques have been widely used to widen the rising edge time of the incident wave or reduce the reverberation time across the specimen, thus facilitating the dynamic mechanical behavior testing of materials like polymers [17], foams [18], muscle [19], and skin [20].

Despite modifications to the split-Hopkinson bar technique for testing the dynamic mechanical properties of soft materials, spike-like stress features still appear in transmitted signals [7,21], differing from quasi–static responses. Researchers have found that spike stress is caused by the radial inertia effect [22], particularly when loading ultra-soft materials like brain tissues and foams [23,24]. Since Kolsky [25] discovered that radial motion and stress were caused by the Poisson effect, the radial inertia effect has been extensively studied. As demonstrated by Davies and Hunter [26], the inertia effect is a function of density, radius, Poisson’s ratio, and strain acceleration. Though the induced stress is of the order of several MPa, it can be significant for ultra-soft materials [19,27], and hence, it is necessary to investigate whether it is an intrinsic behavior of a material or an experimental error induced by the radial inertia effect. The radial inertia effect is distributed in a parabolic pattern along the radial direction of the sample, and using annular samples can effectively reduce it [10,21,23]. However, many other factors affect the radial inertia effect, and their coupling effects need further investigation.

In this study, a spilt-Hopkinson pressure bar was modified for testing ultra-soft materials. The initial spike-like stress feature in the stress history was investigated first, and the coupling factors were studied and verified through combined experiments and numerical simulations. Finally, methods to reduce the radial inertia effect were proposed and verified. This study provides a research basis for the accuracy of SHPB for the dynamic mechanical performance testing of ultra-soft materials, and the influence of coupling factors on the radial inertia effect offers valuable references for the design and preparation of ultra-soft material specimens.

## 2. Experiments and Simulations

### 2.1. Modified SHPB

The stress–strain response of ultra-soft materials often shows obvious spike-like stress features during the dynamic loading of the SHPB, which is primarily caused by inertia effects [21], and this phenomenon was also found in the experimental research of our work. In this study, lung tissue was introduced to investigate the radial inertia effect on the mechanical behavior of ultra-soft materials. Due to its extreme soft mechanic characteristic, the two difficulties faced were the detection of weak transmitted signals and achieving dynamic stress equilibrium loading on the specimen. Traditional SHPB fails to fully break through them, and thus, modifications to the technique are required.

A polymethyl methacrylate (PMMA) tube with a low elastic modulus prevailed as a transmitted bar in detecting weak transmitted signals from lung tissue with extremely low wave impedance. A PMMA pad with a diameter of 16 mm and a thickness of 5 mm was cemented on the clamping end of the transmitted tube to achieve uniaxial compression loading on the specimen. Semiconductor strain gauges with a high gain factor were selected to be cemented on the transmitted tube 170 mm away from the specimen/transmitted bar interface. The type of semiconductor strain gauge used was SB5-120-P-3, and the sensitivity coefficient was 110; the resistance was 120 Ω; and the elongation percentage was 2%. They were collaboratively employed to amplify the weak transmitted signal to make it detectable.

**Figure 1:**
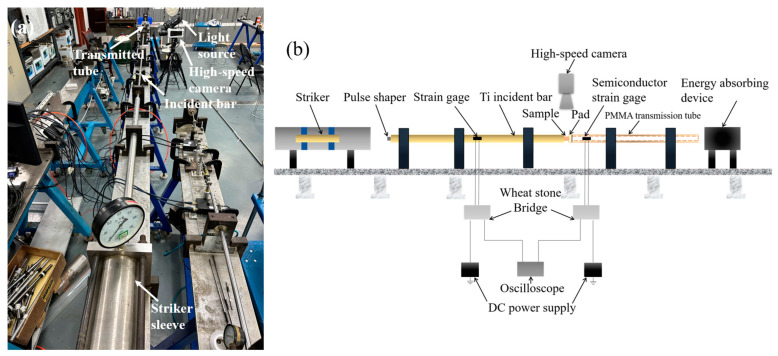
(**a**) The diagram of SHPB experimental setup, and (**b**) the schematic diagram of SHPB experimental setup.

In this study, the striker and incident bar were titanium alloy bars with the same diameter (16 mm) and different lengths (600 mm and 2000 mm, respectively). The specimen impact speeds were 6.2 m/s, 4.1 m/s, and 2.5 m/s, respectively. The duration of the incident pulse T generated in the SHPB experiment is related to the length L of the striker, and T = 2L/C, where C is the elastic wave speed of the striker [28]. Metal grid strain gauges were cemented onto the middle of the incident bar. The type of metal grid strain gauge used was ZF1000-3AA(11)T2-X (which was from No.66 Zhongyuan Road, Puzhen, Hanzhong 723007, Shaanxi, China), and the sensitivity coefficient was 1.92. The resistance was 1000 Ω, and the elongation percentage was 2%. The transmitted tube was a PMMA tube with an outer diameter of 16 mm and an inner diameter of 12 mm, and the length was 1500 mm. The metal grid strain gauges on the incident bar and the semiconductor strain gauges on the transmitted bar were connected to the Wheatstone bridge, respectively. The Wheatstone bridge used for strain measurement in this study is a half-bridge circuit mode. The voltage signal was recorded using an Odyssey high-frequency data acquisition device, and the data sampling rate was set at 10 MHz. A high-speed camera was used to record the deformation process under dynamic loading, with a sampling rate of 64,000 pps and an exposure time of 10 μs, respectively. Since the diameter of the sample was a variable to be considered in experiments, only the 2 mm thickness of the sample was constant here. The modification of thin specimens can reduce the stress wave reverberation time across the specimen and make the specimen achieve stress equilibrium faster. The diagram of the SHPB experimental setup is shown in Figure 1.

**Figure 2:**
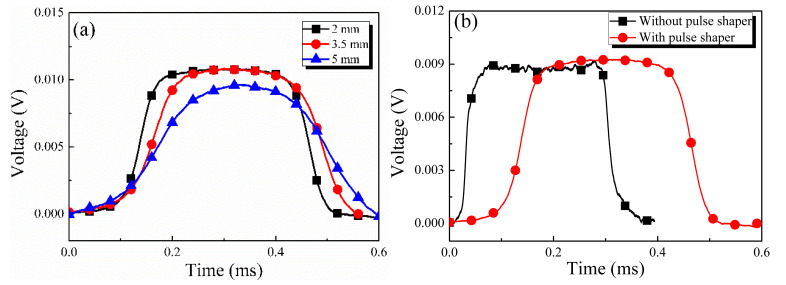
(**a**) Shaping effects of rubber sheets with different lengths; (**b**) the differences with and without pulse shaping technique.

The pulse shaping technique was used in the experiments to widen the rising edge of the incident pulse; the shaping effect of different pulse shapers was tested (see Figure 2 in reference [1] for details), and the rubber sheet was a good shaper. In addition, the shaping effects of rubber sheets with different lengths were tested, which are 2 mm, 3.5 mm, and 5 mm, respectively. The test results indicated that although using rubber sheets with lengths of 3.5 mm and 5 mm can facilitate obtaining incident waves with wider rising edges, there was mutual cancellation between the incident and reflected waves, which seriously affected the experimental results, as shown in Figure 2a. Therefore, the rubber sheet with dimensions of 2 mm × 2 mm × 1 mm was selected as the pulse shaper. In Figure 2b, the incident wave without the pulse shaping technique had a steep rising edge and severe high-frequency oscillation, which affected the experimental results. After using a shaper, the high-frequency component of the stress wave was filtered out, a smooth incident wave signal was obtained in the experiment, and the pulse shaping technology widened the rising edge of the incident wave effectively. The stress equilibrium process can be verified by calculating and comparing the forces of bars/specimen interfaces, which are expressed by Equations (1) and (2) below.

*F_is_(t) = A_i_E_i_(ε_i_(t) +ε_r_(t))* (1)

*F_ts_(t) = A_t_E_t_ε_t_(t)* (2)

where *F_is_* and *F_ts_* are the forces of the incident bar/specimen interface and the transmitted bar/specimen interface, *A_i_* and *A_t_* are the cross-sectional areas of the incident and transmitted bar, respectively, *E_i_* and *E_t_* are the Young’s modulus of incident and transmitted bar, respectively, and *ε_i_*(*t*), *ε_r_*(*t*), and *ε_t_*(*t*) are the incident, reflected, and transmitted strains, respectively.

**Figure 3:**
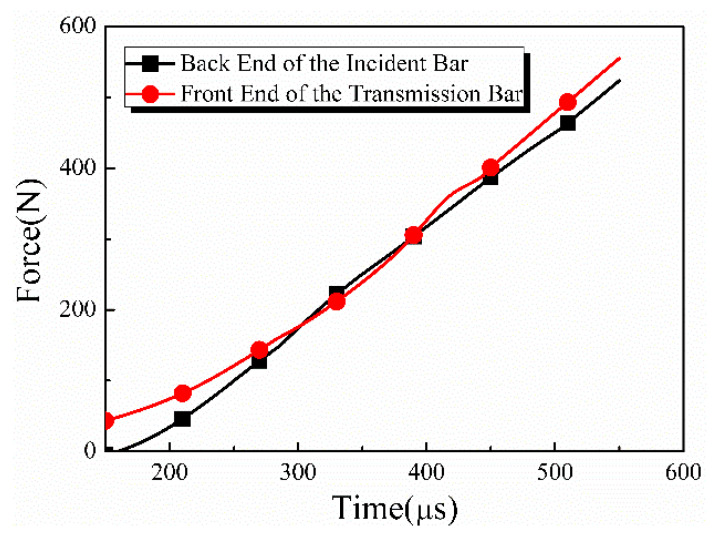
The force histories of bar/specimen interfaces under stress equilibrium state.

**Figure 4:**
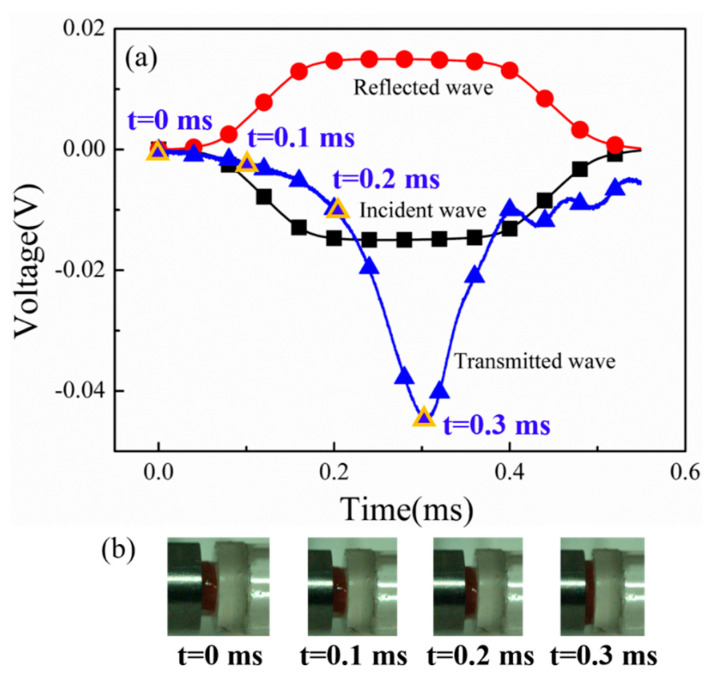
(**a**) The incident, reflected, and transmitted waves aligned in time, and (**b**) the high-speed camera images at different time moments.

According to Equations (1) and (2), the stress histories for the incident bar/specimen and transmitted bar/specimen interfaces, respectively, were calculated, and the resulting curves of stress equilibrium are shown in Figure 3. The good agreement between the two curves indicated that the stress histories at both interfaces of the specimen were approximately the same at 150 μs, and the specimen had achieved a stress equilibrium during dynamic loading after the rising edge of the incident wave. In addition, it can be seen from the high-speed images in Figure 4b that the specimen deformed uniformly during loading.

According to one-dimensional stress wave theory, the engineering strain rate, strain, and stress of the sample are interpreted by the following formulas, which are further obtained according to reference [13].

(3) $$\dot{\varepsilon }=\frac{C_{i}\left[\varepsilon_{i}\left(t\right)-\varepsilon_{r}\left(t\right)\right]-C_{t}\varepsilon_{t}(t)}{L_{s}}$$

(4) $$\varepsilon =\frac{1}{L_{s}}\int_{0}^{t}{[C}_{i}(\varepsilon_{i}\left(t\right)-\varepsilon_{r}\left(t\right))-C_{t}\varepsilon_{t}(t)]dt$$

(5) $$\sigma =\frac{A_{t}}{A_{s}}E_{t}\varepsilon_{t}(t)$$

where *A_t_* and *E_t_* are the cross-sectional area and Young’s modulus of the transmitted tube, respectively; *C_i_* and *C_t_* are the wave speeds of the incident bar and the transmitted tube; *A_s_* and *L_s_* are the initial cross-sectional area and specimen length, and *ε_i_*(*t*), *ε_r_*(*t*), and *ε_t_*(*t*) are the incident, reflected, and transmitted strains, respectively.

The experimental method proposed in this study replaced the traditional SHPB transmitted bar material with PMMA, whose strength was about 100 MPa. The proposed method can effectively test the mechanical properties of ultra-soft materials with a strength less than that of PMMA, such as biological soft tissues, gels, and polymers, whose strengths are at the kPa level or on the order of dozens of MPa.

### 2.2. Sample Preparation

In this study, rabbit lungs were taken as the research object, which were from the First Affiliated Hospital of Xi’an Jiaotong University, Shaanxi Province, China. When preparing the samples, a surgical scalpel, thickness gauge, caliper, and circle blades were used. The common SHPB sample shapes are cube, cuboid, and cylinder, and the sample shapes do not affect the experimental results. In order to facilitate sampling, cylindrical samples were used as the research basis in this study. When cutting the sample, a cylindrical sample was prepared first with a sharp circle blade with a diameter of 10 mm. The thickness of the sample was determined to be 2.5 mm using a thickness gauge. Then, the thickness of the sample was accurately adjusted to be 2.0 mm using a surgical scalpel, and the thickness was measured three times on average at any position with a caliper to ensure the accuracy of the sample size. The sample was then placed in a circular template of the same thickness as the sample, and the inner circle blade was aligned with the template to ensure concentricity and punch a circular hole with a diameter of 5 mm. In order to ensure the uniformity and consistency of the sample, the trachea and blood vessels were avoided when cutting the connective tissue sample.

### 2.3. Characteristic of Spike Stress

**Figure 5:**
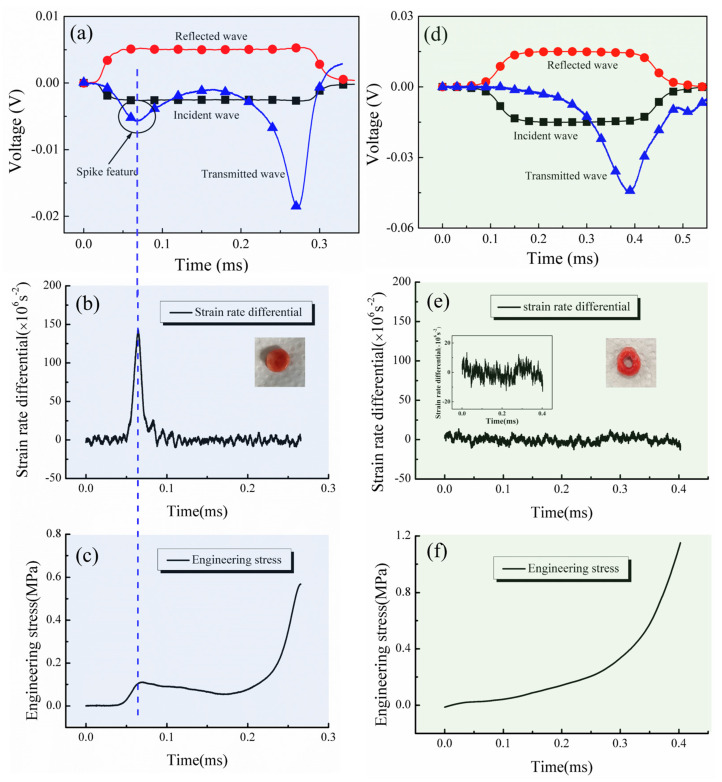
(**a**) The incident, reflected, and transmitted signals of SHPB test on cylindrical lung tissue specimens. The relationship between the strain rate differential and stress history of cylindrical (**b**,**c**) and lung tissue specimens. (**d**) The incident, reflected, and transmitted signals of SHPB test on annular lung tissue specimen. The relationship between the strain rate differential and stress history of annular (**e**,**f**) lung tissue specimen.

Figure 5a shows the original voltage signal of lung tissue loaded by the modified SHPB, where the transmitted signal exhibited a distinct spike marked with a black circle. This phenomenon is also noted in references [2,21]. In order to verify factors potentially contributing to this spike stress, experiments were conducted on two types of specimens. The first one was a cylindrical specimen with a diameter of 10 mm and a thickness of 2 mm. In this study, the strain rate differential was defined as the production of strain rate over time, i.e., strain acceleration [21]. As depicted in Figure 5b,c, the strain rate differential of the cylindrical specimen was displayed along with its stress history, revealing a clear corresponding correlation between the acceleration history and the small spike in the stress history, as marked by the blue dash line. The abrupt increase in the strain rate differential resulted in a spike-like stress feature at the initial part of the stress history, followed by a relatively stable strain rate differential, corresponding to a typical stress–strain curve, as shown in Figure 5c. The second type of specimen was an annular specimen with an outer diameter of 10 mm, an inner diameter of 5 mm, and a thickness of 2 mm. Figure 5d illustrates experimental signals where the spike feature declined in the transmitted signal. The corresponding strain rate differential did not increase abruptly. Meanwhile, the spike stress caused by the radial inertia effect was minimal, as demonstrated in Figure 5e,f. Thus, the cylindrical ultra-soft specimen demonstrated a tendency towards a stable strain rate differential and minimal spike stress. Both experimental results indicated a strong correlation between spike stress and specimen shape, where different shapes produced varying strain rate differentials and affected spike stress characteristics differently.

### 2.4. Finite Element Model

**Figure 6:**
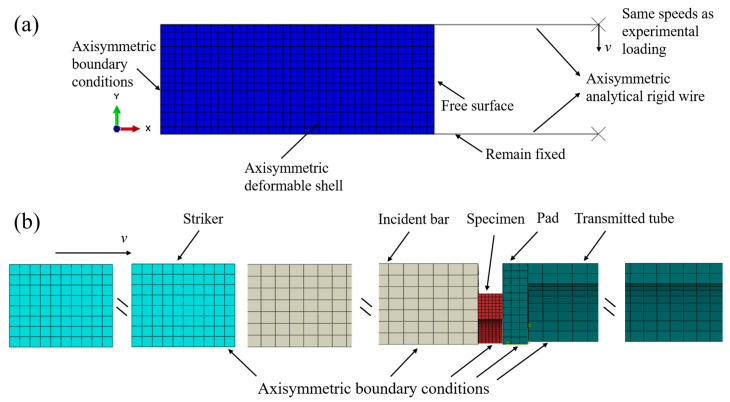
The finite element models and boundary conditions of simplified SHPB (**a**) and complete SHPB (**b**).

**Figure 7:**
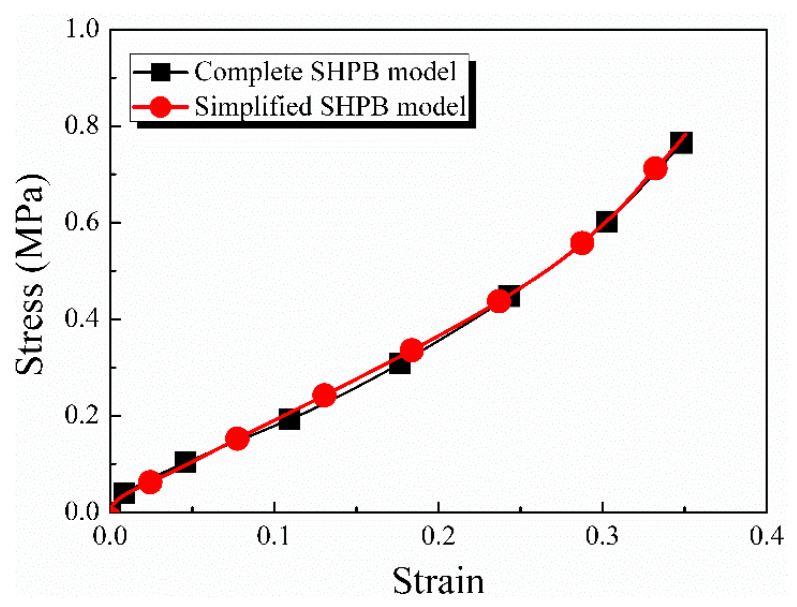
The simulation results of the complete SHPB model and simplified SHPB model.

A finite element analysis was carried out to analyze the influence of materials, geometric parameters, and strain rate differentials on the spike features, and the commercial finite element software package Abaqus 6.14 was used to build the model. Since the lung tissue strength was extremely lower than that of the incident and transmitted bars, the incident bar and transmitted bar were considered two rigid surfaces to sandwich and load the specimen for computational efficiency, as indicated in Figure 6a. Axisymmetric boundary conditions were applied on the left side of the specimen, and the right side was a free surface. The upper rigid surface was applied at the same speed as experimental loading, and the lower one remained fixed. To demonstrate the validity of assuming bars as rigid surfaces, a complete SHPB model was also established based on experimental conditions. The dimensions and material properties of the striker, incident bar, and transmitted tube in the model were the same as those in the experiment. Please refer to Section 2.1 for specific parameters. To enhance computational efficiency, a quarter model was established for the simulation work, with axisymmetric boundary conditions applied to ensure accuracy. The radial velocity and angular velocity of the model were constrained to be zero. And the simulation results were compared with the simplified SHPB model under the same loading condition, which showed the validity of the simplified SHPB model, as shown in Figure 7. The specimen’s constitutive behavior was modeled as homogeneous, isotropic, and hyper-elastic, and the specific material properties are shown in Equation (4) and in Table 2 in reference [29]. The four variable coupling parameters are presented in italics in Table 1, namely the inner diameter, density, Poisson’s ratio, and specimen strain rate differential, respectively. The model details and parameters are listed in Table 1. Therefore, the number of models to be investigated in this study was 6 × 3 × 4 × 3, totaling 216. The total simulation process lasted for 600 μs, which was sufficient to capture incident, reflected, and transmitted waves. Stress and strain histories were obtained by simulating the propagation of the pulse in the specimen.

**Figure 8:**
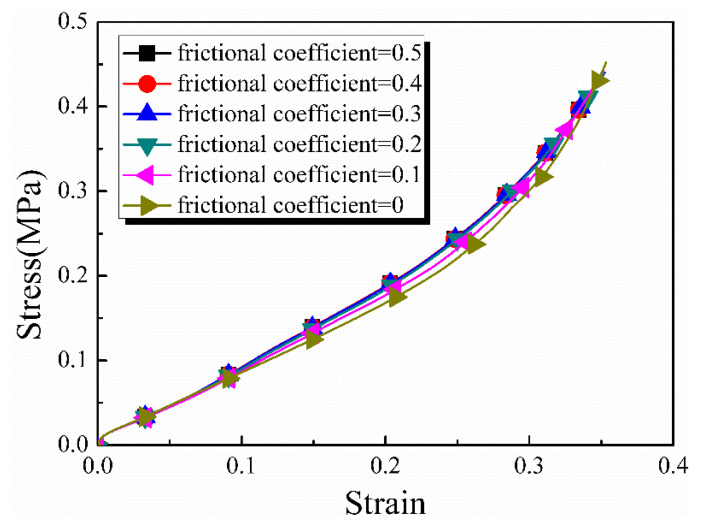
The influence of different friction coefficients on the stress strain curves.

To assess the effect of friction between the specimen ends and the rigid surfaces, simulations were conducted with varying friction coefficients; references [30,31] suggest that the friction coefficient between metals should generally be between 0.5 and about 0.3 for the interface between polymers and metals. The lung, as a biological soft tissue material, had tissue fluid on the surface. Additionally, Vaseline was applied to the bar clamping ends to minimize friction; therefore, a friction coefficient of 0.5 was taken as the maximum critical value. The results in Figure 8 indicate that the friction coefficients were nearly in good agreement, with minimal influence observed from different friction coefficients during the dynamic loading process. Therefore, a numerical analysis was performed with a frictionless assumption on the interfaces in the subsequent work.

## 3. Results and Discussion

**Figure 9:**
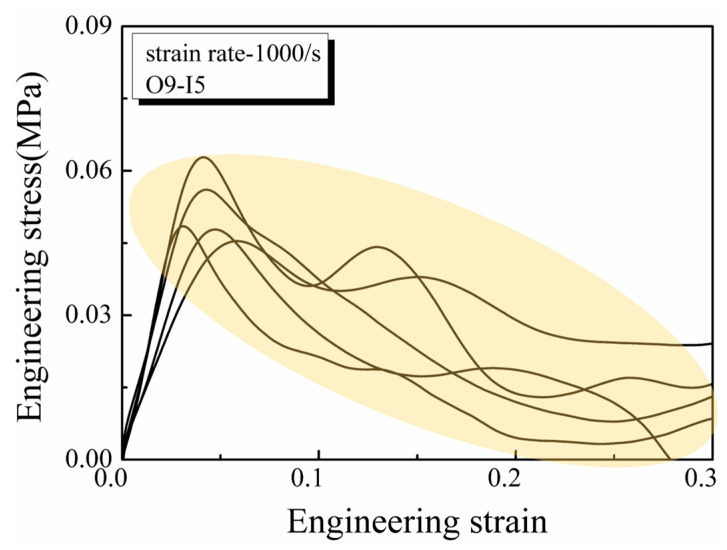
Experimental repeatability of SHPB tests on lung tissues. All specimens have an outer diameter of 9 mm and an inner diameter of 5 mm. Strain rate 1000 s^−1^ corresponds to a strain rate differential of 0.7 × 10^8^ s^−2^.

In this study, experimental investigations were performed with a focus on experiment repeatability. The yellow area in Figure 9 illustrates the coincident mechanical behaviors observed during compression tests on lung tissue at a loading rate of 1000 s^−1^, demonstrating the repeatability and suitability of our approach for soft materials.

**Figure 10:**
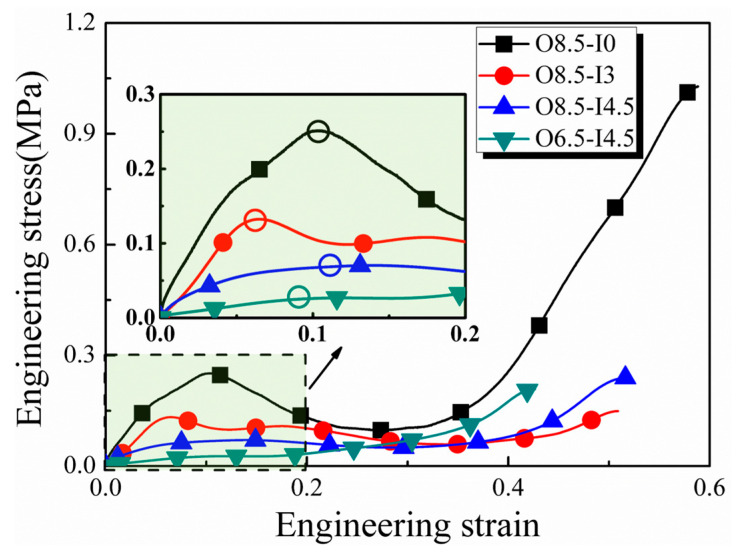
Stress histories of lung tissues with various outer and inner diameters. The hollow circles mark the peak spike stresses.

The influence of specimen diameter on spike stress was examined through a series of experiments, performing various inner diameters with a constant outer diameter and strain acceleration. As shown in Figure 10, “O” represents the outer diameter, and “I” is the inner diameter. Under a nearly constant strain rate differential of 1.3 × 10^8^ s^−2^ and a constant strain rate of ~2000 s^−1^, we observed that larger inner diameters corresponded to lower spike stresses. With respect to the specimen with the same inner diameter and strain acceleration, the larger the outer diameter, the higher the spike stress. The experimental results indicated that under the same strain rate differential, the magnitude of spike stress was affected by the ratio of inner and outer diameters, hereinafter defined as the diameter ratio. Notably, when this ratio exceeded 50%, spike stress was significantly reduced.

**Figure 11:**
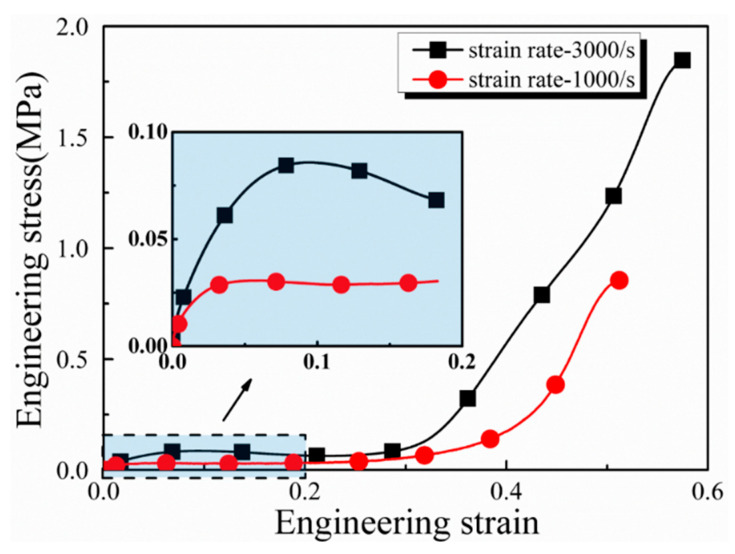
Stress histories of lung tissues under different strain rate differentials. The inner and outer diameters of the specimens are 3 mm and 6.5 mm. Strain rates of 3000 s^−1^ and 1000 s^−1^ correspond to strain rate differentials of 2.5 × 10^8^ s^−2^ and 0.7 × 10^8^ s^−2^.

For specimens with identical inner and outer diameters, variations in radial inertia stresses were determined by different initial strain rate differentials. As depicted in Figure 11, with respect to the specimen with the outer and inner diameters of 6.5 mm and 3 mm, the radial inertia stress at the initial strain rate differential of 2.5 × 10^8^ s^−2^ was higher than that at 0.7 × 10^8^ s^−2^. This stress increased nearly proportionally with a higher strain rate differential, so it was concluded that both the diameter ration and strain rate differential were primary factors influencing the radial inertia effect.

**Figure 12:**
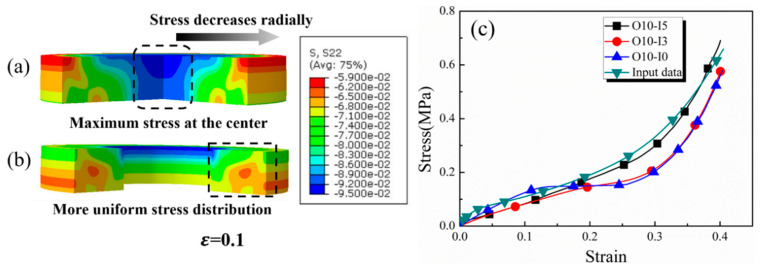
The meshed finite element model at ε = 0.1 of solid specimen (**a**) and annular specimen (**b**); only three quarters of the model is shown for viewing. (**c**) The input data and stress–strain numerical simulation data under different diameter ratios at Poisson’s ratio 0.3.

In order to elucidate the influence of multiple coupling factors, a series of simulation studies were conducted, and the results are discussed below. Firstly, a finite element simulation was used to verify the measurement accuracy of the modified equipment and method. Figure 12a,b illustrate that the stress distribution in the cylindrical specimen with a diameter of 10 mm and a thickness of 2 mm was non-uniform, with the maximum stress occurring at the center and gradually decreasing radially. In contrast, the stress distribution in the annular specimen with an outer diameter of 10 mm, an inner diameter of 5 mm, and a thickness of 2 mm appeared to be more uniform, highlighting the advantages of the annular configuration. The density of both specimens is 550 kg/m^3^, and the strain rate differential is 0.7 × 10^8^ s^−2^. To study the reasonable specimen size, the different sizes of the specimens were simulated and compared with the input experimental data. The simulation findings in Figure 12c revealed that among specimens of varying dimensions, those with an outer diameter of 10 mm and an inner diameter of 5 mm exhibit stress curves closest to the input data. This underscored the benefits of the optimized specimen size, which subsequently guided the following work.

**Figure 13:**
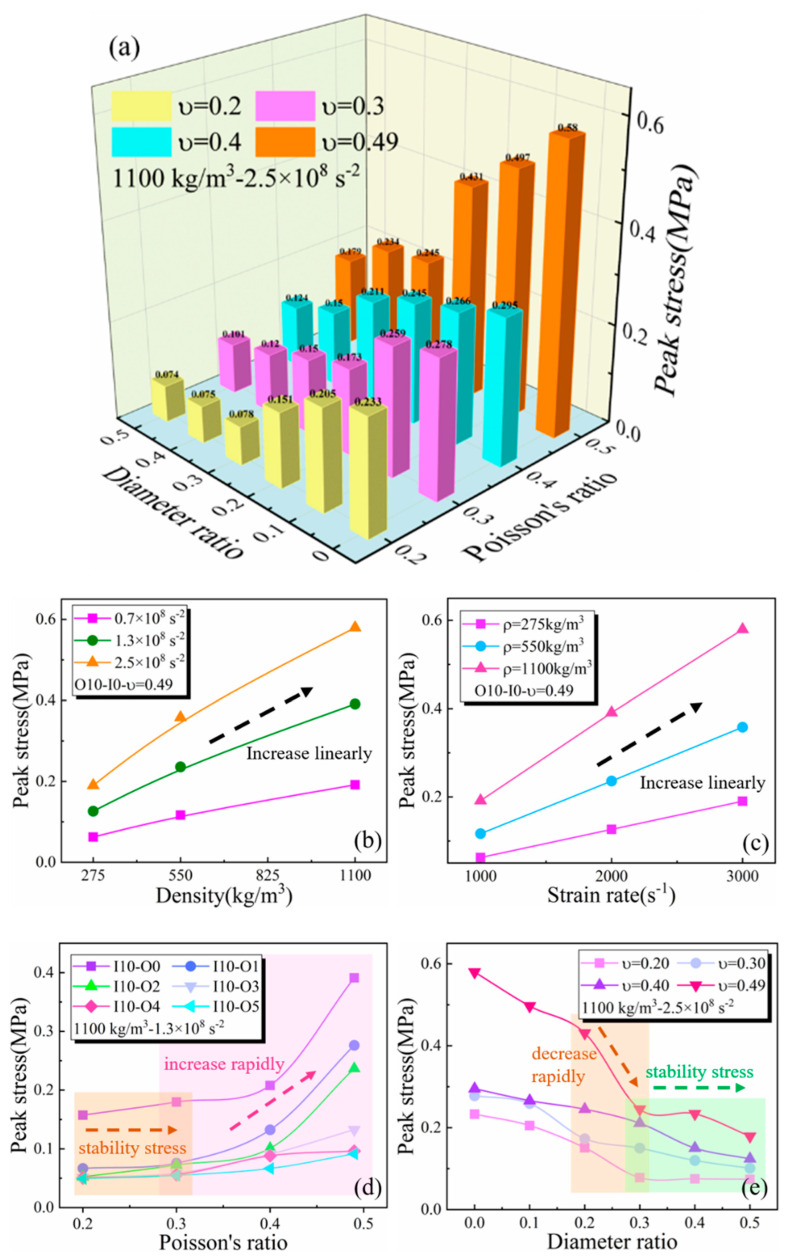
(**a**) The peak spike stress changes with the Poisson’s ratio and diameter ratio at 2.5 × 10^8^ s^−2^ strain rate differential and density of 1100 kg/m^3^. The correlation of peak spike stress with density (**b**) and strain rate (**c**), namely the specimen with a 10 mm outer diameter and 0 mm inner diameter and a Poisson’s ration of 0.49, is taken as an example. (**d**) Simulation results of peak spike stress under various Poisson’s ratios, a density of 1100 kg/m^3^, and a strain rate differential of 1.3 × 10^8^ s^−2^. (**e**) Simulation results of peak spike stress under various diameter ratios, a density of 1100 kg/m^3^, and a strain rate differential of 2.5 × 10^8^ s^−2^.

To investigate the coupling effect of multiple factors on the radial inertia effect, four factors were involved, including the diameter ratio, Poisson’s ratio, strain rate differential, and density in finite element simulation analysis. Figure 13a displays the simulation results at a strain rate differential of 2.5 × 10^8^ s^−2^ and a density of 1100 kg/m^3^. The trend in the simulation results was the same at the other two strain rate differentials and densities, except that the amplitude increased proportionally with the increase in strain rate differentials and densities; duplicate figures for other conditions were omitted for brevity. The results show that peak axial spike stress varied with the various diameter ratios, Poisson’s ratios, and densities when the strain rate differentials were 0.7 × 10^8^ s^−2^, 1.3 × 10^8^ s^−2^, and 2.5 × 10^8^ s^−2^, corresponding to strain rates of 1000 s^−1^, 2000 s^−1^, and 3000 s^−1^, respectively.

The peak stress of the initial spike increased proportionally with density and strain rate differential, as shown in Figure 13b,c. Therefore, the peak spike stress was linearly dependent on the density and strain rate differentials of the specimen. The peak stress was not only related to the density but also to three other coupling factors. Linear curve fitting between the densities was only meaningful for discussion when the other three factors remained determined; otherwise, linear curve fitting between densities was not deterministic. For the split-Hopkinson bar experiments of biological soft tissues, the initial strain acceleration typically reached around 10^8^ s^−2^, assuming a sample diameter of 10 mm, material density of 1000 kg/m^3^, and Poisson’s ratio of 0.45, and the additional axial stress was about 2.5 MPa. For metal materials or even polymer materials, the additional axial stress caused by the inertia effect was negligible. However, for ultra-soft materials with strengths in MPa and kPa, the stress–strain history exhibited prominent spike features, resulting in significant measurement inaccuracies.

**Figure 14:**
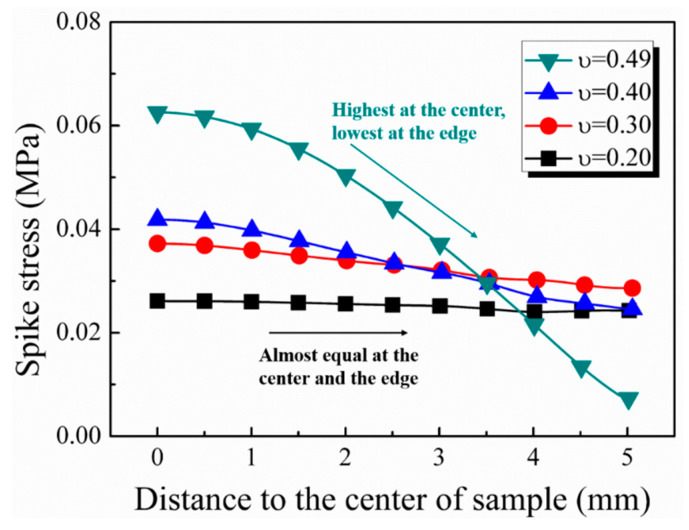
Peak spike stress at different locations from the center of the sample under different Poisson’s ratios.

Figure 13d demonstrates that the peak spike stress increased with higher Poisson’s ratios, stabilizing when the ratio fell below 0.3. In addition, as shown in Figure 14, when the Poisson’s ratio was below 0.3, there was little difference between the spike stresses at the center and the edge of the specimen, and the spikes during the dynamic loading of the specimen were no longer obvious. In order to further illustrate the influence of Poisson’s ratio on the radial inertia effect, considering the extreme case where the material’s Poisson’s ratio was 0, no matter how soft the material was, the radial inertia effect would not occur and thus affect the experimental results. Simulation results also verified this conclusion that the specimen exhibited no spike features and responded nearly linearly elastic with a Poisson’s ratio of 0. Conversely, spike stress increased rapidly when the Poisson’s ratio exceeded 0.3. It is thus concluded that the spike feature was caused by radial deformation under dynamic loading, which decreased when the radial deformation of the specimen was not severe.

Case studies were conducted using specimens with various Poisson’s ratios, a density of 1100 kg/m^3^, and a strain rate differential of 2.5 × 10^8^ s^−2^ to analyze the relationship between the peak spike stress and the diameter ratio. The specimen with a zero diameter ratio was a cylindrical specimen, and the results in Figure 13e show that the radial inertia effect of the cylindrical specimen was particularly obvious under all Poisson’s ratio cases, and thus, the peak spike stress was the highest. When the diameter ratio exceeded 20%, the peak rapidly decreased, plateauing beyond 30%. Notably, for all Poisson’s ratio cases, the radial inertia stress was minimal at a diameter ratio of 0.5, aligning closely with the experimental findings shown in Figure 12c.

**Figure 15:**
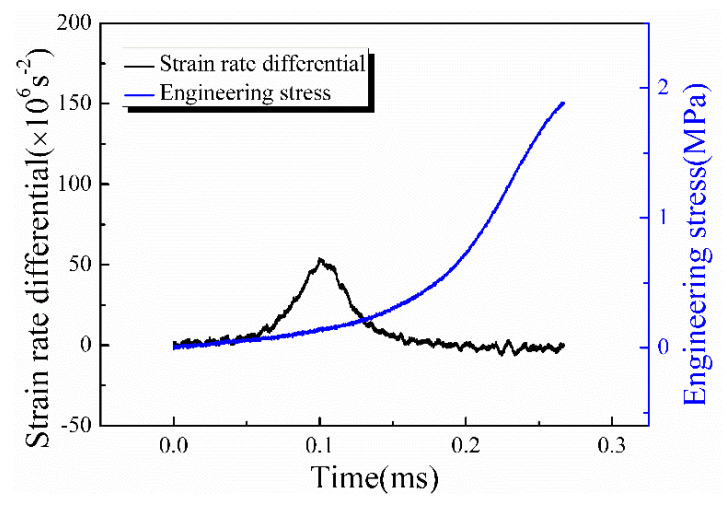
The strain rate differential and stress history with pulse shaping technique when the strain rate is 3000 s^−1^; the ordinate is consistent with Figure 5b if comparing the amplitude of the strain rate differential.

In the actual experiments, the density and Poisson’s ratio of the material were usually regarded as nearly constant, while the outer diameter of the specimen was constrained by the bar diameter of the modified apparatus. Therefore, the inner diameter of the specimen and the strain rate differential played important roles in the magnitude of spike stress. Consequently, two methods are proposed to mitigate the axial spike stress caused by the radial inertia effect: altering the specimen’s geometry and reducing the strain rate differential during initial loading. The results depicted in Figure 14 revealed that the spike stress was distributed in a parabolic trend, with the peak stress occurring at the specimen center. Therefore, removing the central part of the specimen to form an annular specimen can greatly reduce the spike stress. Additionally, the pulse shaping technique can diminish the strain rate differential by widening the rising edge of the incident stress wave. An unshaped incident pulse contained a high-frequency component, which caused the strain acceleration in the specimen and led to the rapid rise in the strain rate differential. In contrast, a pulse shaper can filter out these high-frequency components through its large deformation, resulting in a smooth incident pulse. Therefore, the initial strain acceleration of dynamic loading can be reduced, thus leading to a decreased strain rate differential. As shown in Figure 15, the strain rate differential at 3000 s^−1^ was 0.5 × 10^8^ s^−2^ with the aid of a pulse shaper, which was one-fifth of the differential without the pulse shaper. In conclusion, the pulse shaping technique proved instrumental in effectively reducing spike stress. 

## 4. Conclusions

This study investigated the causes of spike-like stress features observed in the stress history obtained from split-Hopkinson bar loading experiments on ultra-soft specimens. The potential factors were verified and analyzed through dynamic experiments and numerical simulations. It was determined that the initial spike stress was caused by the radial inertia effect. Finally, methods to mitigate the radial inertia effect were proposed. The experimental results of this study provide a research basis for testing the dynamic mechanical properties of biological tissue, polymers, and other ultra-soft materials and advance industrial fields such as constitutive models, impact defense, and the rapid diagnosis of organisms. Some conclusions are drawn and listed below.

1. The peak spike stress varied linearly with the density and strain acceleration. When the Poisson’s ration exceeded 0.3, it was distributed in a parabolic trend along the radial direction of the specimen; this trend diminished when the ratio was less than 0.3.
2. The simulation results indicate a direct relationship between the radial inertia stress and the diameter ratio of the specimen. The radial inertia stress decreased significantly when the diameter ratio exceeded 20% and stabilized above 30%. As for the diameter ratio of 50%, the minimum radial inertia stress occurred, and the results were preferred, which was also confirmed due to their good agreement with experimental results.
3. Two methods were proposed to reduce the spike stress caused by the radial inertia effect. One was removing the central part of the specimen to form an annular specimen, and the other was filtering the high-frequency component of the incident stress wave via the pulse shaping technique.

The above-concluded results could serve as guidance for the research of the radial inertia effect, the analysis of additional stress spikes, and even an experimental reference for ultra-soft materials.

## Figures and Tables

**Table 1:** The parameters of the finite element models.

*Inner Diameter* (mm)	Outer Diameter (mm)	Length (mm)	*Density* (kg/m^3^)	*Poisson’s Ratio*	*Strain Rate Differential* (s^−2^)	Elements Types
0 1 2 3 4 5	10	2	275 550 1100	0.2 0.3 0.4 0.49	0.7 × 10^8^ 1.3 × 10^8^ 2.5 × 10^8^	4-node bilinear CAZ4R

## Data Availability

The original contributions presented in the study are included in the article, further inquiries can be directed to the corresponding author.
